# Preventive Mechanism of Lycopene on Intestinal Toxicity Caused by Cyclophosphamide Chemotherapy in Mice by Regulating TLR4-MyD88/TRIF-TRAF6 Signaling Pathway and Gut-Liver Axis

**DOI:** 10.3390/nu14214467

**Published:** 2022-10-24

**Authors:** Xiao Pan, Xiaoyan Niu, Yaping Li, Yupei Yao, Lirong Han

**Affiliations:** Key Laboratory of Public Health Safety of Hebei Province, Ministry of Education, College of Public Health, Hebei University, Baoding 071002, China

**Keywords:** lycopene, cyclophosphamide, intestinal microbiota, gut-liver axis, TLR4-MyD88/TRIF-TRAF6 signaling pathway

## Abstract

Cyclophosphamide (CYC) is the first-line chemotherapy drug for cancer in clinical practice, and its intestinal toxicity seriously affects the treatment effect and prognosis of patients. Lycopene (LP) is the main pigment of ripe tomatoes and has strong antioxidant activity. However, the mechanism by which LP prevents CYC-induced intestinal injury remains unclear. The aim of this study was to investigate the mechanism of LP in preventing intestinal toxicity caused by CYC chemotherapy in mice. The results showed that LP significantly prevented spleen and thymus atrophy induced by CYC. In terms of intestinal injury, LP significantly increased the levels of superoxide dismutase (SOD), secretory immunoglobulin A (sIgA), interleukin (IL)-4, IL-12, and interferon (IFN)-γ, decreased the content of lipid oxidation (MDA), upregulated the protein expressions of toll-like receptors 4 (TLR4), myeloid differentiation factor 88 (MyD88), tumor necrosis factor receptor-associated factor 6 (TRAF6), toll/IL-1receptor domain containing adaptor protein inducing IFN-β (TRIF), *p*-P38 MAPK (P38), and *p*-nuclear factor kappa-B (NF-κB) p65, and improved the small intestine tissue injury induced by CYC. In terms of liver injury, LP significantly increased the content of glutathione (GSH), decreased the contents of MDA, nitric oxide (NO), IL-1β, IL-6, and tumor necrosis factor (TNF)-α, and repaired the liver tissue injury induced by CYC. Importantly, 10 mg/kg LP significantly prevented intestinal microbiota dysregulation in CYC mice. These results suggested that LP significantly prevented intestinal injury induced by CYC in mice by regulating the TLR4-MyD88/TRIF-TRAF6 signaling pathway and gut-liver axis.

## 1. Introduction

Cyclophosphamide (CYC) was the first so-called “latent” broad-spectrum anticancer drug developed in the 1950s, which was approved by the Food and Drug Administration for treating leukemia and solid tumors, mostly in combination with other anticancer drugs [1]. Due to its precise efficacy and low price, CYC has become the main drug in first-line chemotherapy for cancer patients. Unfortunately, CYC has various toxic side effects in use, among which the gastrointestinal side effects are the most obvious, such as vomiting, diarrhea, gastrointestinal bleeding, and other serious complications. During chemotherapy with CYC, nausea and vomiting occurred in more than 30% of patients, and diarrhea occurred in about 10~30% of patients [2]. Some researchers conducted an early investigation on breast cancer patients treated with CYC chemotherapy and found that nausea and fatigue were the “most troublesome” side effects that patients considered [3]. Studies have shown that CYC causes intestinal microbiota imbalance, leading to immune dysfunction and weakened barrier function, so intestinal microbiota homeostasis plays an important role in the development and maintenance of the immune system and the intestine. Intestinal immune function impairment caused by CYC is an important cause of secondary ulcerative colitis, irritable bowel syndrome, and acute pancreatitis, which seriously affects the prognosis and quality of life of patients [4]. In addition, liver injury is also one of the main toxic side effects of CYC [5]. CYC must be transformed into activated phosphoramide mustard by the liver in vivo to play an anticancer role. Various substances that reduce the hepatotoxicity of CYC are associated with their reduction in oxidative stress [6]. Therefore, using antioxidants reduces CYC-induced oxidative stress. The liver and intestine regulate each other and are closely linked. Marshall proposed the concept of the “gut-liver axis” in 1998; the intestinal mucosal barrier was damaged after an intestinal attack, and a large number of intestinal bacteria and lipopolysaccharide were transferred to the liver through the portal vein system, which promoted the release of inflammatory factors in the liver, further causing intestinal and distant organ damage [7]. Liver diseases are closely related to intestinal microbiota, and intestinal inflammation and imbalance of bacterial microbiota promote the formation of liver diseases [8]. Intestinal microbiota regulates metabolism through the gut-liver axis and is closely related to liver inflammation and hepatocellular carcinoma [9]. Thus, a complex network of interactions and influences among various cells, inflammatory factors, and metabolites in the gut-liver axis is formed [10].

Currently, there is no specific drug for intestinal toxicity caused by CYC, and antiemetic drugs, such as metoclopramide and ondansetron, are mostly used clinically to alleviate this injury [11]. However, these drugs also cause adverse reactions and increase the burden on cancer patients. Therefore, how to safely and effectively prevent intestinal toxicity caused by CYC and improve the quality of life of cancer patients is particularly important. Compared with drug prevention and treatment, nutritional intervention is not only an effective method to prevent and control the toxicity of chemotherapy drugs, but also the natural nutrients are safe and non-toxic, which can help improve the quality of life of patients [12]. Recently, studies on the toxic side effects of CYC have become increasingly in-depth, mainly focusing on the toxicity of immune function, liver, reproductive, and renal toxicity and intervention [6]. At present, relevant studies mainly focus on the intestinal toxicity caused by CYC intervention by polysaccharides [13]. Polysaccharides interfering in CYC-induced intestinal toxicity mainly include modulation of the intestinal microbiota, secretory immunoglobulin A (sIgA), tight-junction-related proteins, and Toll-like receptors 4 (TLR4)/mitogen-activated protein kinases (MAPKs)/ nuclear factor kappa-B (NF-κB) signaling pathway [14]. However, due to the large variety, large molecular weight and complex structure of polysaccharides, it is difficult to clearly analyze the structure–activity relationship [15]. The intake of high-dose polysaccharides also leads to a decrease in intestinal microbiota diversity [16]. Therefore, it is urgent to find new substances to prevent intestinal injury caused by CYC and open up new ideas for its clinical nutrition prevention and treatment.

Lycopene (LP) is a natural carotenoid with strong antioxidant activity, mainly found in agricultural vegetables and fruits, such as tomatoes, watermelon, grapefruit and carrots [17]. The molecular formula of LP is C_40_H_56_, and the relative molecular weight is 536.85. LP is a deep red fat-soluble pigment, which is widely used as a food colorant or health food. LP cannot be synthesized in the human body and can only be obtained through diet, which has various health benefits and no harmful effects have been found in high doses [18]. Therefore, researchers are increasingly interested in the physiological functions of LP, which are antioxidant, anti-inflammatory, immunomodulatory, anticancer, and antitumor [19,20]. It is worth noting that the antioxidant capacity of LP is 100 times that of vitamin E, due to the conjugated double bond contained in the structure [21]. Stojiljkovic et al. [22] found that LP avoided oxidative damage of cells by stabilizing cell membranes or scavenging free radicals, thus playing a protective role in methotrexate-induced kidney damage in rats. In addition, Rajput et al. [23] showed that 10 mg/kg LP had a protective effect on intestinal epithelial injury induced by deoxynienol. The main mechanism was that LP upregulated tight-junction proteins, increased intestinal beneficial bacteria, antioxidant enzyme activities, intestinal villus height, and crypt depth, and regulated the Keap1/Nrf2 signaling pathway to alleviate oxidative damage of intestinal epithelial cells. Our previous study also found that LP prevented UC in DSS-induced ulcerative colitis mice by inhibiting antioxidant activity, blocking the TLR4/toll/IL-1receptor domain-containing adaptor proteins inducing IFN-β (TRIF)/NF-κB signaling pathways, and repairing tight junctions [24]. However, there are few reports on whether LP prevents intestinal immune function injury caused by CYC, and its mechanism is unclear. In this study, by establishing a mouse model of CYC-induced intestinal injury, the mechanism of LP in preventing intestinal immune function damage by regulating the gut-liver axis was investigated by detecting mouse organ index, intestinal mucosal antioxidant capacity and antibody secretion, morphological structure of the liver and small intestine tissue, cytokine secretion, intestinal microbiota changes, and TLR4-myeloid differentiation factor88 (MyD88)/TRIF-tumor necrosis factor receptor-associated factor 6 (TRAF6) signaling pathways. This study aims to provide a new way for alleviating intestinal damage caused by CYC, and lay a foundation for the wide application of LP.

## 2. Materials and Methods

### 2.1. Materials

LP (≥98%, 20 mg) was purchased from Solarbio (Bejing, China), and CYC (1 g) from Supelco (Sigma-Aldrich, St. Louis, MO, USA). The kits of total superoxide dismutase (SOD) activity, lipid oxidation (MDA), nitric oxide (NO), BCA protein concentration, and glutathione (GSH) were purchased from Beyotime (Shanghai, China). The sIgA ELISA kit was purchased from Elabscience Biotechnology (Wuhan, China). The interleukin (IL)-4, IL-12, interferon (IFN)-γ, IL-1β, IL-6, and tumor necrosis factor (TNF)-α ELISA kits were purchased from Beyotime (Shanghai, China). TLR4, MyD88, TRAF6, TRIF, *p*-P38 MAPK (P38), P38, *p*-NF-κB p65, NF-κB p65, β-actin, and horseradish peroxidase-conjugated secondary antibodies were purchased from Beyotime (Shanghai, China). The hematoxylin-eosin (HE) staining kit was purchased from Beyotime (Shanghai, China). All other chemicals were of the analytically purest grade available.

### 2.2. Methods

#### 2.2.1. LP Intragastric Dose Selection and Preparation

Excessive intake of LP does not cause toxic side effects in humans, so there is no international recommended daily intake for LP. In this study, the low, medium, and high doses of LP were set to 5 mg/kg·bw, 10 mg/kg·bw, and 20 mg/kg·bw, respectively, by referring to pre-experimental dose screening results. LP was prepared and diluted with olive oil, and the gavage volume was 5 mL/kg·bw. LP final concentrations in low-, medium-, and high-dose groups were 1 mg/mL, 2 mg/mL, and 4 mg/mL, respectively.

#### 2.2.2. Preparation of Animal Models

Sixty healthy C57BL/6 mice aged 6–8 weeks (male and female in half) were selected, clean grade, 18–22 g, which were purchased from Sibeifu Biotechnology Co., LTD (Beijing, China). All animal procedures were performed in accordance with the Guidelines for Care and Use of Laboratory Animals of “Hebei University” and approved by Animal Ethical and Welfare Committee. Mice were randomly divided into normal group, CYC group, low-dose LP (5 mg/kg·bw) + CYC group, medium-dose LP (10 mg/kg·bw) + CYC group, and high-dose LP (20 mg/kg·bw) + CYC group, with 12 mice in each group. LP treatment groups were administered with the corresponding dose of LP solution (0.1 mL/d). The normal group and CYC group were administered olive oil (0.1 mL/d) for 30 days. On day 15 to 17, except for the normal group, the other groups were intraperitoneally injected with 0.1 mL/d cyclophosphamide (70 mg/kg·bw) to establish the intestinal injury model of mice. The normal group was injected with the same amount of normal saline.

#### 2.2.3. Visceral Index

During the whole experiment period, the basic condition of the mice was observed daily and their weight was recorded. After fasting for 16 h (free drinking water), the mice were sacrificed and their spleen and thymus were weighed and recorded. Spleen and thymus indexes are calculated as follows:

$$\mathrm{Spleen}\;\mathrm{index}=\mathrm{Spleen}\;\mathrm{weight}\;\left(\mathrm{mg}\right)/\mathrm{Body}\;\mathrm{weight}\;\left(g\right)\times 10$$

$$\mathrm{Thymus}\;\mathrm{index}=\mathrm{Thymus}\;\mathrm{weight}\;\left(\mathrm{mg}\right)/\;\mathrm{Body}\;\mathrm{weight}\;\left(g\right)\times 10$$

#### 2.2.4. Detection of Biochemical Indicators of the Small Intestine

The small intestine tissues were taken and 10% tissue homogenate was prepared according to the mass/volume ratio of the tissue to the pre-cooled normal saline at 1:10. Centrifugation was performed at 3000 r/min for 15 min. The supernatants were collected and the levels of MDA, SOD, IL-4, IL-12, and IFN-γ in small intestine tissues were determined strictly according to the kit instructions.

#### 2.2.5. Detection of sIgA Content in Intestinal Mucosa

The middle jejunum and ileum were, respectively, cut off, placed on ice, cut longitudinally along the intestine, and washed with pre-cooled PBS. Part of ileum mucosa and jejunum mucosa were scraped, diluted at 1:1 and centrifuged at 3000 r/min at 4 °C for 10 min. The content of sIgA in intestinal mucosa was determined by the ELISA kit using supernatants, and the specific steps were carried out in strict accordance with the instructions.

#### 2.2.6. Western Blot

The small intestine tissues were homogenized and centrifuged, and the protein concentrations were determined by the BCA method. The protein expression levels of TLR4, MyD88, TRAF6, TRIF, *p*-P38, and *p*-NF-κB p65 in small intestinal tissues were determined by Western Blot, as described in Han et al. [25].

#### 2.2.7. Detection of Biochemical Indicators of the Liver

The liver tissues were prepared into 10% homogenate, centrifuged at 3000 r/min for 15 min, and the supernatant was collected. The levels of MDA, GSH, NO, IL-1β, IL-6, and TNF-α in the liver tissues were determined strictly according to the kit instructions.

#### 2.2.8. Determination of Intestinal Microbiota

Illumina PE300 sequencing platform was used to detect and analyze the changes of intestinal microbiota in mice by 16S rRNA gene sequencing. According to the primer sequence (338F: ACTCCTACGGGAGGCAGCAG; 806R: GGACTACHVGGGTWT CTAAT), V3-V4 variable region-specific amplification of fecal DNA and bacterial 16S rRNA sequencing. After obtaining sequencing data, optimized sequence and taxonomic analysis, diversity analysis, dilution curve analysis, sample grouping analysis, community composition analysis, and significant difference analysis between populations were performed to evaluate the diversity of intestinal microbiota.

#### 2.2.9. Statistical Analysis

SPSS 24.0 statistical software (Armonk, New York, NY, USA) was used for statistical analysis. All count data were expressed as mean ± standard deviation (SD). Analysis of variance was used for the comparison between groups and within groups, and *p* < 0.05 was considered a significant difference level.

## 3. Results

### 3.1. Effects of LP on Spleen and Thymus Indexes in CYC Mice

As shown in Table 1, compared with the normal group, spleen and thymus indexes of mice in the CYC group were significantly decreased (*p* < 0.01), indicating that intraperitoneal injection of CYC caused spleen and thymus atrophy in mice. Compared with the CYC group, the spleen and thymus indexes of mice in the LP treatment group were increased to varying degrees, and the effect of the M-LP group was the most significant (*p* < 0.05, *p* < 0.01), indicating that LP effectively prevented the atrophy and injury of immune organs in mice caused by CYC.

### 3.2. Effects of LP on Intestinal Immune Function Injury Induced by CYC in Mice

#### 3.2.1. Effects of LP on SOD, MDA, sIgA, and Cytokine Levels in CYC Mice Intestinal Tissue

**Figure 1:**
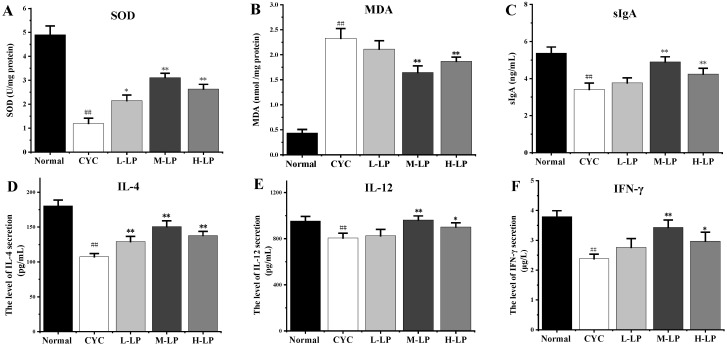
Effects of LP on SOD (**A**), MDA (**B**), sIgA (**C**), IL-4 (**D**), IL-12 (**E**), and IFN-γ (**F**) in intestinal tissues of mice. Note: *n* = 3, ^##^ *p* < 0.01 vs. Normal group, * *p* < 0.05, ** *p* < 0.01 vs. CYC group.

As shown in Figure 1A,B, compared with the normal group, the SOD activity in the small intestinal tissues of mice in the CYC group was significantly decreased (*p* < 0.01), while the MDA content was significantly increased (*p* < 0.01). However, after pretreatment with different doses of LP, the SOD activity in the small intestine tissues of mice was increased and the MDA content was decreased to different degrees, among which M-LP had the most significant effect (*p* < 0.01). Figure 1C showed that, compared with the normal group, the intestinal mucosal sIgA secretion level of mice in the CYC group was significantly decreased (*p* < 0.01). After pretreatment with different doses of LP, the secretion level of sIgA in the intestinal mucosa of mice increased to varying degrees, and that in the M-LP group was significantly increased (*p* < 0.01). As shown in Figure 1D–F, compared with the normal group, CYC treatment significantly inhibited the secretion levels of IL-4, IL-12, and IFN-γ in the small intestine of mice, and the levels of all cytokines increased in different degrees after pretreatment with different doses of LP, among which 10 mg/kg LP had the most significant effect (*p* < 0.01).

#### 3.2.2. Effects of LP on Small Intestinal Morphology in CYC Mice

**Figure 2:**
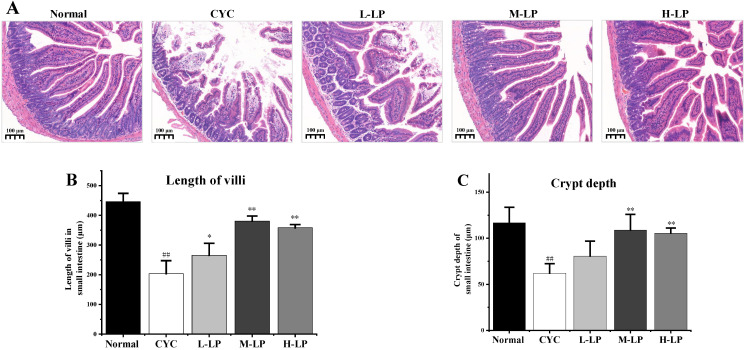
Effects of LP on histological morphology of small intestine in mice. (**A**) HE staining of small intestine (10×); (**B**) Length of villi in the small intestine; (**C**) Crypt depth of small intestine. Note: *n* = 3, ^##^ *p* < 0.01 vs. Normal group, * *p* < 0.05, ** *p* < 0.01 vs. CYC group.

The histopathological observation of the small intestine was shown in Figure 2A. In the normal group, the villi of the small intestine were orderly arranged, with deep crypts and thick and clear brush borders. While in the CYC group, the lengths of the villi in the small intestine were significantly decreased, the crypts were shallow or absent, the intestinal wall was thinned, there was slight edema, and some cells had inflammatory infiltration. After the administration of different doses of LP, the intestinal-related structures and morphology of mice in the CYC group were improved to different degrees. The improvement of intestinal tissue structure in the M-LP group was more obvious than that in other LP treatment groups. The improvement of intestinal tissue structure in the M-LP group was more obvious than that in other LP treatment groups. In addition, as shown in Figure 2B,C, the villus length and crypt depth of the small intestine in the CYC group were significantly decreased compared with the normal group (*p* < 0.01). Compared with the CYC group, the length of the intestinal villus in LP groups was increased, especially in the M-LP group (*p* < 0.01). In addition, the crypt depths of the small intestine were also increased in LP groups, especially in M-LP group (*p* < 0.01).

#### 3.2.3. Effects of LP on Key Proteins in Small Intestine of CYC Mice

**Figure 3:**
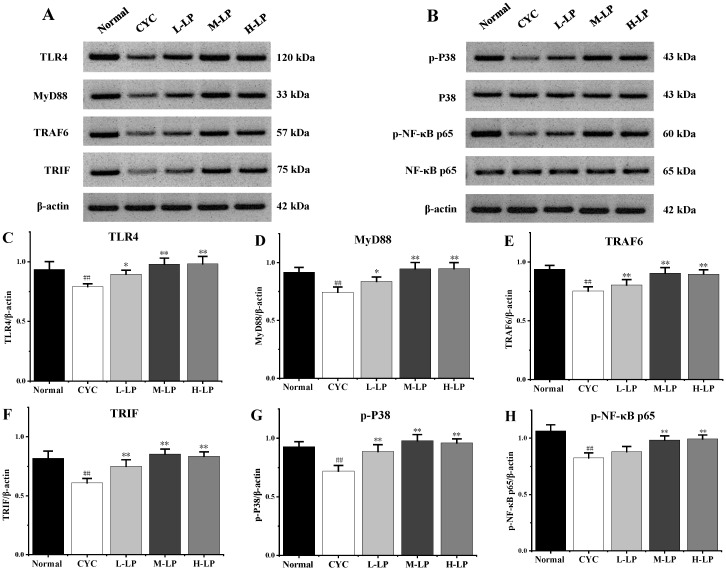
Effects of LP on the key proteins in small intestine of mice. (**A**) Western blot bands of TLR4, MyD88, TRAF6, and TRIF. (**B**) Western blot bands of *p*-P38, P38, *p*-NF-κB p65, and NF-κB p65. (**C**–**H**) Quantitative analysis of TLR4, MyD88, TRAF6, TRIF, *p*-P38, and *p*-NF-κB p65, respectively. Note: *n* = 3, ^##^ *p* < 0.01 vs. Normal group, * *p* < 0.05, ** *p* < 0.01 vs. CYC group.

As shown in Figure 3A–H, CYC significantly downregulated the protein expression levels of TLR4, MyD88, TRAF6, TRIF, *p*-P38, and *p*-NF-κB p65 in small intestinal tissues, compared with the normal group (*p* < 0.01). Compared with the CYC group, these protein expression levels were upregulated in different doses after pretreatment with LP, and 10 mg/kg LP was the most significantly upregulated (*p* < 0.01).

### 3.3. Effects of LP on CYC-Induced Liver Injury in Mice

**Figure 4:**
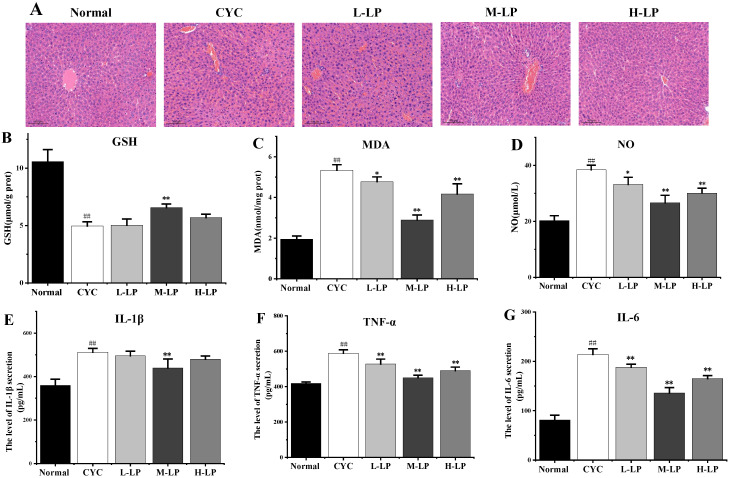
Effects of LP on CYC-induced liver injury in mice. (**A**) Effects of LP on morphology and structure of liver tissue in mice (20×). (**B**–**G**) Effects of LP on GSH (**B**), MDA (**C**), NO (**D**), IL-1β (**E**), TNF-α (**F**), and IL-6 (**G**) in mouse liver tissues. Note: *n* = 3, ^##^ *p* < 0.01 vs. Normal group, * *p* < 0.05, ** *p* < 0.01 vs. CYC group.

The results of HE staining of the liver are shown in Figure 4A. The liver cells in the normal group were arranged regularly, and no abnormal pathological changes were found. In the CYC group, the original normal liver tissue structure was destroyed, the hepatic cord arrangement was disordered, the hepatic lobule was blurred, the boundary was not clear, the liver cells were edema, and there were a lot of irregular vacuoles. In the low-dose LP group, the liver cells were swollen and cloudy, the interfacial boundary was unclear, and there were still a few inflammatory cells infiltrating and necrotic foci. The structures of most liver cells in medium- and high-dose LP groups were more intact than that in the CYC group, and the cell infiltration induced by inflammation was significantly improved, the boundary between liver cells was clearer, the cytoplasm was slightly mixed, and only a few vacuoles were found.

As shown in Figure 4B–D, compared with the normal group, the contents of MDA and NO in liver tissue in the CYC group were significantly increased (*p* < 0.01), and the content of GSH was significantly decreased (*p* < 0.01). Compared with the CYC group, the contents of MDA and NO in medium- and high-dose LP groups were significantly decreased (*p* < 0.01), and the content of GSH in the M-LP group was significantly increased (*p* < 0.01).

As shown in Figure 4E–G, IL-1β, TNF-α, and IL-6 contents were significantly increased compared with the normal group (*p* < 0.01). Compared with the CYC group, the contents of TNF-α and IL-6 were significantly decreased after LP treatment (*p* < 0.01). The IL-1β content in the M-LP group was significantly decreased (*p* < 0.01), but there was no statistical significance in low- and high-dose LP groups.

### 3.4. Effects of LP on Intestinal Microbiota Induced by CYC in Mice

**Figure 5:**
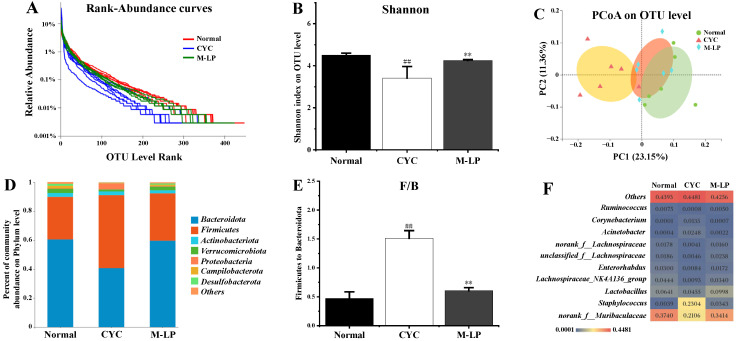
Effect of LP on CYC-induced intestinal microbiota imbalance in mice. (**A**) Rank-abundance curves; (**B**) Shannon index; (**C**) PCoA analysis; (**D**) Analysis of intestinal microbiota structure at phylum level; (**E**) F/B ratio; (**F**) Analysis of differential intestinal microbiota at genus level. Note: *n* = 6, ^##^ *p* < 0.01 vs. Normal group, ** *p* < 0.01 vs. CYC group.

Medium-dose LP (10 mg/kg) was selected, and 16S rRNA sequencing was used to analyze the changes in intestinal microbiota in each group. A total of 47,640 high-quality bacterial 16S rRNA gene reads were obtained by Illumina high-throughput gene sequencing. According to sequence similarity greater than 97%, 593 ± 24 OTUs were obtained in each group. As shown in Figure 5A, rank-abundance curve results showed that the normal group curve was the largest in the X-axis range, indicating that the normal control group had the highest species abundance of intestinal microbiota, while the CYC group curve was the smallest in the X-axis range, with the lowest species abundance. The M-LP group was close to the normal group, indicating that the 10 mg/kg LP treatment restored the decrease in species abundance caused by CYC to a certain extent. As shown in Figure 5B, at the OUT classification level, compared with the normal group, the Shannon index of the CYC group was significantly decreased (*p* < 0.01), indicating that the diversity of intestinal microbiota of mice was significantly reduced after CYC treatment. After intragastric treatment with 10 mg/kg LP, the Shannon index was significantly increased (*p* < 0.01), indicating that the intestinal microbiota diversity of mice recovered. As shown in Figure 5C, PCoA analysis results showed that there was a significant separation between the normal group and the CYC group, and the M-LP group moved to the normal group, indicating that LP had a certain regulatory effect on the composition of intestinal microbiota and close to the normal group. As shown in Figure 5D,E, at the phylum level, *Bacteroidetes* and *Firmicutes* were the two most dominant phylum in the three groups, accounting for more than 90% of the total number of bacteria. Compared with the normal group, the ratio of *Firmicutes* to *Bacteroidota* (F/B) in the CYC group was significantly increased (*p* < 0.01), while 10 mg/kg LP treatment reduced the F/B ratio (*p* < 0.01). As shown in Figure 5F, at the genus level, compared with the normal group, the CYC group significantly reduced the relative abundance of beneficial bacteria (norank_f__Muribaculaceae, Lactobacillus, Lachnospiraceae_NK4A136_group, Enterorhabdus, unclassified_f__Lachnospiraceae, norank_f__Lachnospiraceae, and Ruminococcus), significantly increased the relative abundance of harmful bacteria (*Staphylococcus*, *Acinetobacter*, and *Corynebacterium*). Compared with the CYC group, LP treatment significantly reversed the above trend, indicating that LP can effectively prevent CYC-induced intestinal microbiota imbalance in mice.

## 4. Discussion

LP, a carotenoid widely present in tomatoes and their products, is a potent natural antioxidant that can be used to prevent human health disorders [26,27]. CYC is a kind of chemotherapy drug commonly used in clinics, which belongs to cycle non-specific drugs and is often used to treat breast cancer, prostate cancer, and other cancers, as well as autoimmune diseases [28]. However, CYC can produce great toxic and side effects during chemotherapy, leading to bone marrow suppression, reproductive toxicity, liver toxicity, renal toxicity, and intestinal toxicity. Patients often discontinue treatment because they cannot tolerate its obvious side effects. Studies have shown that CYC inhibits cellular and humoral immunity of the body, and also causes damage to immune organs such as bone marrow, thymus, and spleen. Therefore, CYC is often used as an immunosuppressant to establish animal immune suppression models. The spleen and thymus are important immune organs of mammals, and their weight changes can directly reflect the changes in the immune system. In many studies, the spleen and thymus indexes are important indexes to evaluate the immune defense ability of the body [29]. In this study, we established a CYC-induced intestinal injury model in mice. Compared with the CYC group, the M-LP group significantly increased spleen and thymus indexes, suggesting that LP effectively prevents spleen and thymus atrophy in mice induced by CYC.

As the largest immune organ, the intestinal tract plays a vital role in the body’s immune function. CYC can damage intestinal epithelial cells, resulting in increased intestinal permeability, impaired intestinal mucosal oxidation, and intestinal microbiota disorder, thus damaging intestinal immune function [30,31]. SOD activity can indirectly reflect the ability of scavenging oxygen free radicals, and MDA can be used to judge the level of oxygen free radicals in the colonic mucosa, the intensity of lipid peroxidation, and the degree of tissue damage [32]. Cai et al. [33] found that alhagi honey polysaccharides significantly improve intestinal SOD activity, reduce MDA content, relieve intestinal oxidative stress injury, and the protect intestinal barrier in CYC mice. In the present study, it was found that LP significantly increased SOD activity and decreased MDA content in small intestinal tissues, suggesting that LP effectively alleviates the oxidative damage of the gut caused by CYC in mice. The sIgA is an important antibody mediating humoral immunity. Fu et al. [34] showed that codon polysaccharide increased the level of ileum-secreted immunoglobulin A (sIgA), thus playing an important role in the protection of intestinal mucosal immune injury caused by CYC. Our results showed that, compared with the CYC group, both medium and high doses of LP significantly increased intestinal mucosal sIgA secretion levels, suggesting that LP effectively restores intestinal mucosal immune function in CYC mice. IL-12 plays an important role in enhancing cellular immunity. IL-4 is secreted by activated Th2 cells and promotes B cell proliferation and activation. IFN-γ is secreted by Th1 cells and enhances cellular immune function. Therefore, the levels of IL-4, IL-12, and IFN-γ cytokines can reflect the status of humoral and cellular immunity [35]. Our results showed that the levels of IL-4, IL-12, and IFN-γ in the CYC group were significantly decreased, indicating that CYC inhibited the cellular and humoral immunity of mice. LP at different doses increased the levels of those cytokines to different degrees, suggesting that LP significantly restores the intestinal immunity of CYC mice. In addition, intestinal tissues HE staining results showed that, compared with the CYC group, medium- and high-dose LP group of the majority of small intestinal villus neatly, deep fossae, intestinal wall thick brush border, and intestinal tissue sIgA and cytokines in the trend of test results is consistent, suggesting that LP significantly prevents and relieves the CYC mice intestinal tissue damage degree, strengthens the intestinal immune response ability.

It is well known that TLRs are pattern recognition receptors linking innate and adaptive immunity, and the signal transduction pathway mediated by TLRs mainly includes two pathways: The MyD88-dependent pathway and TRIF pathway (also known as MyD88-independent pathway) [36]. The MyD88-dependent pathway exists in most TLR signaling pathways, and the TRIF pathway mainly exists in TLR3 and TLR4 signaling pathways. MyD88 is a key downstream signal ligand of the TLR4 receptor complex and an important adaptor protein of NF-κB signaling pathway. TRAF6 is a key signal transduction protein downstream of MyD88, which induces the activation of NF-κB and regulates downstream gene expressions. TLR4 further activates TRAF6 through the MyD88-dependent pathway, activating the NF-κB signaling pathway, thereby regulating the expression of immune or inflammatory genes [37]. In addition, TLR4 can activate TRAF6 by regulating TRIF, and then induce the expression of cytokines such as IL-1, IL-4, IL-8, IL-12, and IFN-γ through the activation of JNK, P38, and other MAPK pathways. Choi et al. [38] found that LP inhibited the expression of inflammatory cytokines by downregulating the expression of TLR4 protein and alleviating oxidative stress injury in respiratory epithelial cells A549 stimulated by house dust mites. Our previous study showed that LP prevented DSS-induced UC in mice by regulating the TLR4/TRIF/NF-κB signaling pathway [24]. In this study, the effect of LP on the prevention of CYC-induced intestinal injury and the mechanism of the TLR4-MyD88/TRIF-TRAF6 signaling pathway were investigated. The results of this study showed that medium- and high-dose LP upregulated the protein expressions of TLR4, MyD88, TRIF, TRAF6, *p*-P38, and *p*-NF-κB p65 in intestinal tissues of CYC mice. Zhao et al. [39] found that polysaccharides from bee-collected pollen of Chinese wolfberry activated the colonic TLR4/MyD88/NF-κB p65 pathway and the intestinal immune response, thus repairing the intestinal injury caused by CYC and protecting the integrity of the intestinal epithelial barrier. Ying et al. [40] showed that *Cordyceps sinensis* polysaccharides activated TLR4/NF-κB by upregulating the protein expressions of TLR-2, TLR-4, TLR-6, *p*-IκB-α, and NF-κB p65, then promote cytokines IL-12, IFN-γ, IL-4, IL-13, IL-6, IL-17, IL-10, TGF-β3, TNF-α, IL-2, and IL-21, thereby alleviating the immunosuppressive state of CYC-induced intestinal mucosa in mice. According to literature reports and our experimental results, it was speculated that the mechanism by which LP regulated cytokines was by activating TLR4 receptors and then regulating the protein expressions of downstream MyD88 and TRIF, respectively, both MyD88 and TRIF further activated TRAF6, TRAF6 further promoted phosphorylation of P38 and NF-κB p65 at the same time, and finally promoted the secretion of related immune cytokines (IL-4, IL-12, and IFN-γ) in intestinal tissues of mice with CYC-induced intestinal injury. These results indicate that LP activates intestinal immunity through the TLR4-MyD88/TRIF-TRAF6 signaling pathway, which plays a role in preventing intestinal injury caused by CYC.

The gut is an important digestive organ and the liver is the most critical metabolic organ. The gut-liver axis plays an important role in the regulation of human health [41]. Intestinal damage will lead to direct exposure of the liver to enterogenic toxins, and liver damage will also lead to intestinal dysfunction. Hepatotoxicity is one of the main toxic side effects of CYC, which can cause reactive oxygen species accumulation in liver cells and lead to cellular oxidative stress, resulting in liver damage [42]. Several antioxidants have been found to reduce CYC-induced oxidative stress damage to the liver [43]. Temel et al. [44] established a rat model of liver injury induced by CYC, and the results showed that in the CYC group, the levels of MDA, alanine transaminase, and alkaline phosphatase in serum increased, leading to obvious liver injury, and the levels of SOD, catalase, and GSH in serum decreased, while chrysin reversed these changes and played a protective role in liver toxicity induced by CYC. LP has powerful antioxidant activity, and its antioxidant capacity is 100 times that of vitamin E [21]. In this study, compared with the CYC group, the contents of MDA and NO in liver tissues of the medium-dose LP group were significantly decreased, while the content of GSH was significantly increased. Oxidative stress plays a similar role as a “second messenger” in an inflammatory response, and inflammatory cytokines such as IL-1β, IL-6, and TNF-α are important physiological messenger molecules in an inflammatory response. A study comparing plasma antioxidant levels in 57 patients with non-alcoholic steatohepatitis (NASH) with healthy controls found that NASH patients had significantly lower plasma levels of tocopherols and carotenoids, and LP was the main carotenoid in human plasma and could be used as an effective lipophilic antioxidant to prevent NASH [45]. Our results found that, compared with the CYC group, LP reduced the contents of TNF-α, IL-1β, and IL-6 in liver tissues, thereby improving the inflammatory response of the liver induced by CYC. In addition, liver HE staining results in this study showed that compared with the CYC group, most liver cells in the medium- and high-dose LP groups were more complete in structure, cell infiltration caused by inflammation was significantly improved, the boundary between liver cells was clearer, the cytoplasm was slightly mixed, and only a few vacuolates were observed. These results suggest that LP is closely related to its antioxidant capacity in preventing CYC-induced liver injury.

The intestinal microbiota is the microbiota that lives in the gut, including bacteria, fungi, and viruses, with bacteria as the predominant one. Under normal circumstances, the intestinal microbiota is symbiotic with the host in a specific way, once the disturbance of the microbiota will cause the body barrier function to weaken, leading to disease. Studies have found that intestinal microbiota can regulate the body’s metabolism through the gut-liver axis and is closely related to liver inflammation. Liver products can affect intestinal microbiota composition and gut barrier function, and gut factors can also affect liver function. Therefore, the gut microbial community is critical for maintaining the homeostasis of the gut-liver axis. Hartmann et al. [8] found that after the intestinal barrier was damaged, intestinal microbiota and their products reach the liver through the portal vein and lead to liver fibrosis in mice through TLR2 signaling of lamina propria monocytes and tumor necrosis factor receptor I signaling pathway of intestinal epithelial cells. Studies have shown that CYC cause dysregulation of intestinal microbiota and impair intestinal immune function in mice. Wang et al. [46] also established an immunosuppressed mouse model using CYC, and found that *Lactiplantibacillus plantarum* JLAU103 extracellular polysaccharides restored intestinal microbiota disorder caused by CYC, thus regulating intestinal immune response and activating systemic immunity. Our results showed that both medium and high doses of LP had a certain preventive effect on CYC-induced intestinal injury in mice, and the prevention effect of the medium-dose group was the most significant. LP has been found to have a good health function and can bring a variety of health benefits, and excess LP has not been found toxic side effects [18]. However, for the body, the beneficial effect of any substance is in the right dose, and more is not always better. In our experiment, it was also found that although the effect of lycopene in the high-dose group was significant, it was not as good as that in the medium-dose group, indicating that lycopene had the optimal dose range for the prevention of CYC-induced intestinal injury. Therefore, the medium-dose LP (10 mg/kg) was selected to analyze the effect of LP on intestinal microbiota composition in CYC-treated mice in this study. Our results showed that LP treatment significantly increased the number of OTUs and α-diversity index in the intestinal microbiota of CYC mice. The Shannon index reflects the diversity of the community, and the higher the index value, the higher the diversity of the community. Our results indicated that LP helps restore the richness of intestinal microbiota in mice treated with CYC. By analyzing and comparing the dominant intestinal microbiota at the phylum level, our results showed that *Bacteroidetes* and *Firmicutes* were the two most dominant phylum, LP significantly increased the relative abundance of *Bacteroidetes* and decrease the relative abundance of *Firmicutes*, which was consistent with previous reports [47], indicating that LP intervention could significantly reduce the F/B ratio.

At the genus level, the *norank_f__Muribaculaceae* bacterial group, regulated by LP, was detected in mouse feces in this study. Studies have shown that *Muribaculaceae* belongs to *Bacteroidetes*, which dominates the intestinal microbiota of mice, and its relative abundance has a strong correlation with propionic acid [48]. In addition, this study found that LP increased the relative abundance of beneficial bacterium groups (*Lactobacillus*, *Lachnospiraceae_NK4A136_group*, *unclassified_f__Lachnospiraceae*, and *norank_f__Lachnospiraceae*). Huang et al. [49] found that CYC destabilized the intestinal microbiota, and sodium alginate significantly increased the abundance of beneficial bacteria (*Lactobacillus* and *Lachnospiraceae* NK4A136) and ultimately protected intestinal mucosal damage, which was consistent with our study results. *Lachnospiraceae*, a SCFAs-producing bacterium, is positively correlated with the production of SCFAs and acidifies the intestinal environment to prevent intestinal damage from pathogenic bacteria [50]. Therefore, the increased abundance of the three *Lachnospiraceae* species in this study could promote the production of SCFAs, indicating that LP had the function of maintaining the intestinal barrier. *Enterorhabdus* is positively correlated with antitumor immune factors [51], can hydrolyze primary bile acids, alleviate metabolic disorders, and protect the intestinal barrier [52]. The present study found that LP reversed the decrease in *Enterorhabdus* abundance in the CYC group, suggesting that LP may prevent intestinal injury by promoting immune factors. Furthermore, this study also found that LP significantly increased the relative abundance of probiotic *Ruminococcus* and significantly decreased the relative abundance of harmful bacteria (*Staphylococcus*, *Acinetobacter*, and *Corynebacterium*) in the intestinal microbiota of CYC mice. *Ruminococcus* could produce acetic acid in the intestine [53], and *Staphylococcus* was negatively correlated with the body weight of CYC mice [54]. Zhao et al. [53] studied the effects of probiotics *Bacillus coagulans* 13,002 (BCS) and fructo-oligosaccharides (FOS) on intestinal microbiota in CYC mice, and the results showed that BCS increased the abundance of probiotic *Ruminococcus*, BCS + FOS inhibited the production of harmful bacteria *Acinetobacter*, *Corynebacterium*, and *Staphylococcus*. Wiese et al. [55] took moderately obese people (30 < BMI < 35 kg/m²) as the research object, and found that LP relieved hepatic inflammatory oxidative damage and enhanced liver metabolism. In addition, LP changed intestinal microbiota in feces of both the elderly and patients with metabolic syndrome, and the relative abundance of beneficial bacteria *Bifidobacterium* and *Lactobacillu* increased, while harmful bacteria *Proteobacteria* decreased. Therefore, this study demonstrated that LP improved intestinal microbiota composition in CYC mice.

**Figure 6:**
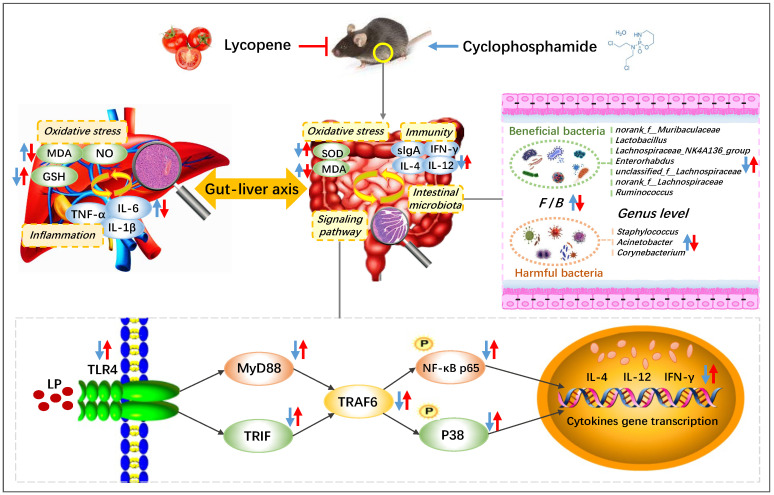
Preventive mechanism of lycopene on intestinal toxicity caused by cyclophosphamide chemotherapy in mice by regulating TLR4-MyD88/TRIF-TRAF6 signaling pathway and gut-liver axis. The blue arrows show the effect of CYC treatment, and the red arrows show the effect of LP treatment on CYC mice. ↑: up-regulation; ↓: down-regulation.

In conclusion, LP improved the spleen and thymus injury of CYC mice to different degrees, effectively promoted the secretion of intestinal immune factors IL-4, IL-12, IFN-γ, and immunoglobulin sIgA, and alleviated the degree of intestinal oxidative injury, regulated the protein expressions of TLR4, MyD88, TRAF6, TRIF, *p*-P38, and *p*-NF-κB p65 in intestinal tissue, increased the content of GSH in liver tissue, reduced the content of MDA, NO, IL-1β, IL-6, and TNF-α, and repaired the liver tissue injury, significantly regulated and normalized the relative abundances of *norank_f__Muribaculaceae*, *Lactobacillus*, *Lachnospiraceae*, *Enterorhabdus*, *Ruminococcus*, *Staphylococcus*, *Acinetobacter*, and *Corynebacterium* (Figure 6). Therefore, LP has an obvious preventive effect on CYC-induced intestinal immune function damage in mice. The main mechanism may be through regulating the gut-liver axis, repairing the intestinal mucosal barrier, activating the TLR4-MyD88/TRIF-TRAF6 signaling pathway to activate the intestinal tract, restoring the diversity of intestinal microbiota, inhibiting the oxidative damage and inflammatory response caused by the transfer of harmful bacteria to the liver, and further playing a preventive role in intestinal immune damage induced by CYC.

## Figures and Tables

**Table 1:** Thymus and spleen indexes of mice in each group.

Groups	Thymus Index (mg/g)	Spleen Index (mg/g)
Normal	2.20 ± 0.33	4.45 ± 0.29
Cyclophosphamide (CYC)	0.93 ± 0.19 ^##^	3.22 ± 0.24 ^##^
Low-lycopene (L-LP)	1.12 ± 0.24	3.39 ± 0.33
Medium-lycopene (M-LP)	1.55 ± 0.15 **	3.88 ± 0.26 *
High-lycopene (H-LP)	1.24 ± 0.15	3.56 ± 0.23

Note: *n* = 3, ^##^ *p* < 0.01 vs. Normal group, * *p* < 0.05, ** *p* < 0.01 vs. CYC group.

## Data Availability

The authors confirm that the data supporting the findings of this study are available within the article.

## Abbreviations

LP	Lycopene
CYC	Cyclophosphamide
sIgA	Secretory immunoglobulin A
TLR4	Toll-like receptors 4
MAPKs	Mitogen-activated protein kinases
NF-κB	Nuclear factor kappa-B
MyD88	Myeloid differentiation factor88
SOD	Superoxide dismutase
MDA	Lipid oxidation
NO	Nitric oxide
GSH	Glutathione
IL	Interleukin
IFN	Interferon
TNF	Tumor necrosis factor
TRAF6	Tumor necrosis factor receptor-associated factor 6
TRIF	Toll/IL-1receptor domain containing adaptor protein inducing IFN-β
P38	p38 MAPK
NASH	Non-alcoholic steatohepatitis
F/B	The ratio of *Firmicutes* to *Bacteroidota*
BCS	*Bacillus coagulans* 13,002
FOS	Fructo-oligosaccharides

## References

[1] Tsuji D., Matsumoto M., Kawasaki Y., Kim Y.-I., Yamamoto K., Nakamichi H., Sahara Y., Makuta R., Yokoi M., Miyagi T. (2021). Analysis of pharmacogenomic factors for chemotherapy-induced nausea and vomiting in patients with breast cancer receiving doxorubicin and cyclophosphamide chemotherapy. Cancer Chemother. Pharmacol..

[2] Emadi A., Jones R.J., Brodsky R.A. (2009). Cyclophosphamide and cancer: Golden anniversary. Nat. Rev. Clin. Oncol..

[3] Sitzia J., Huggins L. (1998). Side effects of cyclophosphamide, methotrexate, and 5-fluorouracil (CMF) chemotherapy for breast cancer. Cancer Pract..

[4] Yang W., Zhao P., Li X., Guo L., Gao W. (2022). The potential roles of natural plant polysaccharides in inflammatory bowel disease: A review. Carbohydr. Polym..

[5] Cengiz M., Yildiz S.C., Demir C., Şahin İ.K., Teksoy Ö., Ayhanci A. (2019). Hepato-preventive and anti-apoptotic role of boric acid against liver injury induced by cyclophosphamide. J. Trace Elem. Med. Biol..

[6] Qian L., Yang F., Lin X., Jiang S., Zhang Y., Tang Y. (2022). Pyrroloquinoline quinone ameliorates liver injury in mice induced by cyclophosphamide. Environ. Sci. Pollut. Res..

[7] Marshall J.C. (1998). The gut as a potential trigger of exercise-induced inflammatory responses. Can. J. Physiol. Pharmacol..

[8] Hartmann P., Haimerl M., Mazagova M., Brenner D.A., Schnabl B. (2012). Toll-like receptor 2-mediated intestinal injury and enteric tumor necrosis factor receptor I contribute to liver fibrosis in mice. Gastroenterology.

[9] Jiang J.-W., Chen X.-H., Ren Z., Zheng S.-S. (2019). Gut microbial dysbiosis associates hepatocellular carcinoma via the gut-liver axis. Hepatobiliary Pancreat. Dis. Int..

[10] Wang S.Z., Yu Y.J., Adeli K. (2020). Role of Gut Microbiota in Neuroendocrine Regulation of Carbohydrate and Lipid Metabolism via the Microbiota-Gut-Brain-Liver Axis. Microorganisms.

[11] Shi Y., He X., Yang S., Ai B., Zhang C., Huang D., Dong M., Liu P., Zhou S., Han X. (2007). Ramosetron versus ondansetron in the prevention of chemotherapy-induced gastrointestinal side effects: A prospective randomized controlled study. Chemotherapy.

[12] Souza A., Silva L., Fayh A. (2021). Nutritional Intervention Contributes to the Improvement of Symptoms Related to Quality of Life in Breast Cancer Patients Undergoing Neoadjuvant Chemotherapy: A Randomized Clinical Trial. Nutrients.

[13] Xie H., Fang J., Farag M.A., Li Z., Sun P., Shao P. (2022). Dendrobium officinale leaf polysaccharides regulation of immune response and gut microbiota composition in cyclophosphamide-treated mice. Food Chem. X.

[14] Li C., Duan S., Li Y., Pan X., Han L. (2021). Polysaccharides in natural products that repair the damage to intestinal mucosa caused by cyclophosphamide and their mechanisms: A review. Carbohydr. Polym..

[15] Li N., Wang C., Georgiev M.I., Bajpai V.K., Qiao X. (2021). Advances in dietary polysaccharides as anticancer agents: Structure-activity relationship. Trends Food Sci. Technol..

[16] Liu F., Li P., Chen M., Luo Y., Prabhakar M., Zheng H., He Y., Qi Q., Long H., Zhang Y. (2017). Fructooligosaccharide (FOS) and galactooligosaccharide (GOS) increase Bifidobacterium but reduce butyrate producing bacteria with adverse glycemic metabolism in healthy young population. Sci. Rep..

[17] Caseiro M., Ascenso A., Costa A., Creagh-Flynn J., Johnson M., Simões S. (2020). Lycopene in human health. LWT.

[18] Grabowska M., Wawrzyniak D., Rolle K., Chomczyński P., Oziewicz S., Jurga S., Barciszewski J. (2019). Let food be your medicine: Nutraceutical properties of lycopene. Food Funct..

[19] Pinto C., Rodriguez-Galdon B., Cestero J.J., Macias P. (2018). Processed tomatoes improves the antioxidant status of carbon tetrachloride-intoxicated rat tissues. Eur. Food Res. Technol..

[20] Abd Al Haleem E.N., Ahmed H.I., El-Naga R.N. (2021). Lycopene and Chrysin through Mitigation of Neuroinflammation and Oxidative Stress Exerted Antidepressant Effects in Clonidine-Induced Depression-like Behavior in Rats. J. Diet. Suppl..

[21] Gajowik A., Dobrzynska M. (2014). Lycopene-antioxidant with radioprotective and anticancer properties. A review. Rocz. Państwowego Zakładu Hig..

[22] Stojiljkovic N., Ilic S., Jakovljevic V., Stojanovic N., Stojnev S., Kocic H., Stojanovic M., Kocic G. (2018). The Encapsulation of Lycopene in Nanoliposomes Enhances Its Protective Potential in Methotrexate-Induced Kidney Injury Model. Oxid. Med. Cell Longev..

[23] Rajput S.A., Liang S.J., Wang X.Q., Yan H.C. (2021). Lycopene Protects Intestinal Epithelium from Deoxynivalenol-Induced Oxidative Damage via Regulating Keap1/Nrf2 Signaling. Antioxidants.

[24] Li Y., Pan X., Yin M., Li C., Han L. (2021). Preventive Effect of Lycopene in Dextran Sulfate Sodium-Induced Ulcerative Colitis Mice through the Regulation of TLR4/TRIF/NF-κB Signaling Pathway and Tight Junctions. J. Agric. Food Chem..

[25] Han L., Yu J., Chen Y., Cheng D., Wang X., Wang C. (2018). Immunomodulatory Activity of Docosahexenoic Acid on RAW264.7 Cells Activation through GPR120-Mediated Signaling Pathway. J. Agric. Food Chem..

[26] Imran M., Ghorat F., Ul-Haq I., Ur-Rehman H., Aslam F., Heydari M., Shariati M.A., Okuskhanova E., Yessimbekov Z., Thiruvengadam M. (2020). Lycopene as a natural antioxidant used to prevent human health disorders. Antioxidants.

[27] Zhao B., Wu J., Li J., Bai Y., Luo Y., Ji B., Xia B., Liu Z., Tan X., Lv J. (2020). Lycopene alleviates DSS-induced colitis and behavioral disorders via mediating microbes-gut–brain axis balance. J. Agric. Food Chem..

[28] Kim J., Chan J.J. (2017). Cyclophosphamide in dermatology. Australas. J. Dermatol..

[29] Han L., Lei H., Tian Z., Wang X., Cheng D., Wang C. (2018). The immunomodulatory activity and mechanism of docosahexenoic acid (DHA) on immunosuppressive mice models. Food Funct..

[30] Xiang X.W., Zheng H.Z., Wang R., Chen H., Xiao J.X., Zheng B., Liu S.L., Ding Y.T. (2021). Ameliorative Effects of Peptides Derived from Oyster (*Crassostrea gigas*) on Immunomodulatory Function and Gut Microbiota Structure in Cyclophosphamide-Treated Mice. Mar. Drugs.

[31] Chen S., Wang J., Fang Q., Dong N., Nie S. (2019). Polysaccharide from natural Cordyceps sinensis ameliorated intestinal injury and enhanced antioxidant activity in immunosuppressed mice. Food Hydrocoll..

[32] Shang L., Liu Y., Li J., Pan G., Zhou F., Yang S. (2021). Emodin Protects Sepsis Associated Damage to the Intestinal Mucosal Barrier Through the VDR/Nrf2/HO-1 Pathway. Front. Pharmacol..

[33] Cai G., Wu Y., Wusiman A., Gu P., Mao N., Xu S., Zhu T., Feng Z., Liu Z., Wang D. (2021). Alhagi honey polysaccharides attenuate intestinal injury and immune suppression in cyclophosphamide-induced mice. Food Funct..

[34] Fu Y.-P., Feng B., Zhu Z.-K., Feng X., Chen S.-F., Li L.-X., Yin Z.-Q., Huang C., Chen X.-F., Zhang B.-Z. (2018). The polysaccharides from Codonopsis pilosula modulates the immunity and intestinal microbiota of cyclophosphamide-treated immunosuppressed mice. Molecules.

[35] Rajanna M., Bharathi B., Shivakumar B., Deepak M., Prabakaran D., Vijayabhaskar T., Arun B. (2021). Immunomodulatory effects of Andrographis paniculata extract in healthy adults–An open-label study. J. Ayurveda Integr. Med..

[36] Tian C., Liu X., Chang Y., Wang R., Yang M., Liu M. (2021). Rutin prevents inflammation induced by lipopolysaccharide in RAW 264.7 cells via conquering the TLR4-MyD88-TRAF6-NF-κB signalling pathway. J. Pharm. Pharmacol..

[37] Duan Y., Dai H., An Y., Cheng L., Shi L., Lv Y., Li H., Wang C., He C., Zhang H. (2022). Mulberry Leaf Flavonoids Inhibit Liver Inflammation in Type 2 Diabetes Rats by Regulating TLR4/MyD88/NF-κB Signaling Pathway. Evid. Based Complement. Altern. Med..

[38] Choi J., Lim J.W., Kim H. (2021). Lycopene Inhibits Toll-Like Receptor 4-Mediated Expression of Inflammatory Cytokines in House Dust Mite-Stimulated Respiratory Epithelial Cells. Molecules.

[39] Zhao Y., Yan Y., Zhou W., Chen D., Huang K., Yu S., Mi J., Lu L., Zeng X., Cao Y. (2020). Effects of polysaccharides from bee collected pollen of Chinese wolfberry on immune response and gut microbiota composition in cyclophosphamide-treated mice. J. Funct. Foods.

[40] Ying M., Yu Q., Zheng B., Wang H., Wang J., Chen S., Nie S., Xie M. (2020). Cultured Cordyceps sinensis polysaccharides modulate intestinal mucosal immunity and gut microbiota in cyclophosphamide-treated mice. Carbohydr. Polym..

[41] Martín-Mateos R., Albillos A. (2021). The role of the gut-liver axis in metabolic dysfunction-associated fatty liver disease. Front. Immunol..

[42] Habibi E., Shokrzadeh M., Chabra A., Naghshvar F., Keshavarz-Maleki R., Ahmadi A. (2015). Protective effects of Origanum vulgare ethanol extract against cyclophosphamide-induced liver toxicity in mice. Pharm. Biol..

[43] Liu Y., Yang A., Qu Y., Wang Z., Zhang Y., Liu Y., Wang N., Teng L., Wang D. (2018). Ameliorative effects of Antrodia cinnamomea polysaccharides against cyclophosphamide-induced immunosuppression related to Nrf2/HO-1 signaling in BALB/c mice. Int. J. Biol. Macromol..

[44] Temel Y., Kucukler S., Yıldırım S., Caglayan C., Kandemir F.M. (2019). Protective effect of chrysin on cyclophosphamide-induced hepatotoxicity and nephrotoxicity via the inhibition of oxidative stress, inflammation, and apoptosis. Naunyn-Schmiedeberg’s Arch. Pharmacol..

[45] Stice C.P., Xia H., Wang X.D. (2018). Tomato lycopene prevention of alcoholic fatty liver disease and hepatocellular carcinoma development. Chronic Dis. Transl. Med..

[46] Wang J., Li M., Gao Y., Li H., Fang L., Liu C., Liu X., Min W. (2022). Effects of Exopolysaccharides from Lactiplantibacillus plantarum JLAU103 on Intestinal Immune Response, Oxidative Stress, and Microbial Communities in Cyclophosphamide-Induced Immunosuppressed Mice. J. Agric. Food Chem..

[47] Xu X., Zhang X. (2015). Effects of cyclophosphamide on immune system and gut microbiota in mice. Microbiol. Res..

[48] Smith B.J., Miller R.A., Ericsson A.C., Harrison D.C., Strong R., Schmidt T.M. (2019). Changes in the gut microbiome and fermentation products concurrent with enhanced longevity in acarbose-treated mice. BMC Microbiol..

[49] Huang J., Huang J., Li Y., Wang Y., Wang F., Qiu X., Liu X., Li H. (2021). Sodium Alginate Modulates Immunity, Intestinal Mucosal Barrier Function, and Gut Microbiota in Cyclophosphamide-Induced Immunosuppressed BALB/c Mice. J. Agric. Food Chem..

[50] Chen X., Sun W., Xu B., Wu E., Cui Y., Hao K., Zhang G., Zhou C., Xu Y., Li J. (2021). Polysaccharides From the Roots of Millettia Speciosa Champ Modulate Gut Health and Ameliorate Cyclophosphamide-Induced Intestinal Injury and Immunosuppression. Front. Immunol..

[51] Zhu H., He Y.S., Ma J., Zhou J., Kong M., Wu C.Y., Mao Q., Lin G., Li S.L. (2021). The dual roles of ginsenosides in improving the anti-tumor efficiency of cyclophosphamide in mammary carcinoma mice. J. Ethnopharmacol..

[52] Han R., Ma Y., Xiao J., You L., Pedisic S., Liao L. (2021). The possible mechanism of the protective effect of a sulfated polysaccharide from Gracilaria Lemaneiformis against colitis induced by dextran sulfate sodium in mice. Food Chem. Toxicol. Int. J. Publ. Br. Ind. Biol. Res. Assoc..

[53] Zhao S., Peng X., Zhou Q.Y., Huang Y.Y., Rao X., Tu J.L., Xiao H.Y., Liu D.M. (2021). Bacillus coagulans 13002 and fructo-oligosaccharides improve the immunity of mice with immunosuppression induced by cyclophosphamide through modulating intestinal-derived and fecal microbiota. Food Res. Int..

[54] Jhang S.Y., Lee S.H., Lee E.B., Choi J.H., Bang S., Jeong M., Jang H.H., Kim H.J., Lee H.J., Jeong H.C. (2021). Effects of Platycodon grandiflorum on Gut Microbiome and Immune System of Immunosuppressed Mouse. Metabolites.

[55] Wiese M., Bashmakov Y., Chalyk N., Nielsen D.S., Krych L., Kot W., Klochkov V., Pristensky D., Bandaletova T., Chernyshova M. (2019). Prebiotic Effect of Lycopene and Dark Chocolate on Gut Microbiome with Systemic Changes in Liver Metabolism, Skeletal Muscles and Skin in Moderately Obese Persons. Biomed. Res. Int..

